# Identification of Paddy Croplands and Its Stages Using Remote Sensors: A Systematic Review

**DOI:** 10.3390/s23156932

**Published:** 2023-08-03

**Authors:** Manuel Fernández-Urrutia, Manuel Arbelo, Artur Gil

**Affiliations:** 1 Departamento de Física, Universidad de La Laguna, 38200 San Cristobal de La Laguna, Spain; manuel.fernandez@ichec.ie (M.F.-U.); marbelo@ull.es (M.A.); 2 Irish Centre for High-End Computing (ICHEC), University of Galway, H91TK33 Galway, Ireland; 3 Research Institute for Volcanology and Risks Assessment (IVAR), University of the Azores (UAc), 9500-321 Ponta Delgada, Portugal

**Keywords:** rice crops, multispectral, multisource, radar, machine learning, vegetation indices

## Abstract

Rice is a staple food that feeds nearly half of the world’s population. With the population of our planet expected to keep growing, it is crucial to carry out accurate mapping, monitoring, and assessments since these could significantly impact food security, climate change, spatial planning, and land management. Using the PRISMA systematic review protocol, this article identified and selected 122 scientific articles (journals papers and conference proceedings) addressing different remote sensing-based methodologies to map paddy croplands, published between 2010 and October 2022. This analysis includes full coverage of the mapping of rice paddies and their various stages of crop maturity. This review paper classifies the methods based on the data source: (a) multispectral (62%), (b) multisource (20%), and (c) radar (18%). Furthermore, it analyses the impact of machine learning on those methodologies and the most common algorithms used. We found that MODIS (28%), Sentinel-2 (18%), Sentinel-1 (15%), and Landsat-8 (11%) were the most used sensors. The impact of Sentinel-1 on multisource solutions is also increasing due to the potential of backscatter information to determine textures in different stages and decrease cloud cover constraints. The preferred solutions include phenology algorithms via the use of vegetation indices, setting thresholds, or applying machine learning algorithms to classify images. In terms of machine learning algorithms, random forest is the most used (17 times), followed by support vector machine (12 times) and isodata (7 times). With the continuous development of technology and computing, it is expected that solutions such as multisource solutions will emerge more frequently and cover larger areas in different locations and at a higher resolution. In addition, the continuous improvement of cloud detection algorithms will positively impact multispectral solutions.

## 1. Introduction

Rice plays a vital role in ensuring food security since it is considered one of the most important crops in the world, feeding nearly 50% of the population. After corn and wheat, paddy rice is considered the third most important staple crop worldwide, but the second overall in production, just behind grain [1]. Extreme weather conditions will likely severely impact rice production [2]. Drought-based events, such as water scarcity [3,4,5,6], and flooding, such as flash flooding, are likely to increase due to climate change and, thus, impact paddy crops [7,8,9,10,11,12,13,14,15,16,17]. Therefore, developing suitable paddy crop monitoring solutions for land management and spatial planning is essential to attenuate the impact of extreme weather events by extracting and assessing pre- and post-event information about paddy fields [4,6,18,19].

**Figure 1:**
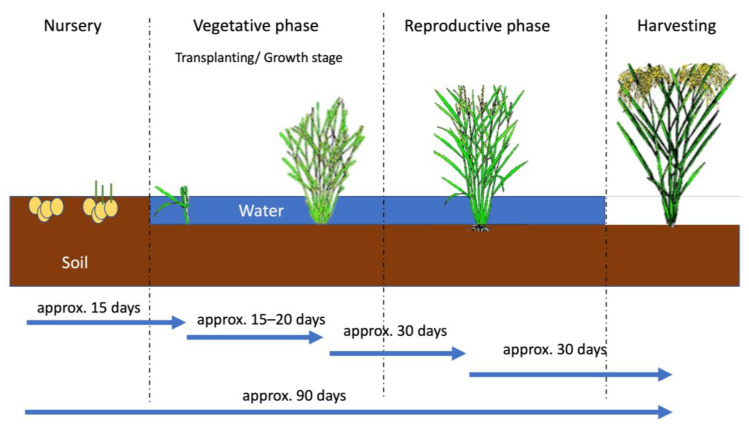
Different growing stages of paddy rice and its approximate timeframe. Paddy images extracted from [21].

However, there is a need for in-depth knowledge in some aspects of physiology, as assessing cultivation stages is essential for monitoring paddy croplands. All crops present different physical characteristics, and paddy rice has different attributes at each growth stage. Four stages are identified during its growth [20]: (a) nursery, (b) vegetative phase, (c) reproductive phase, and (d) harvesting. As shown in Figure 1, the process from sowing to harvest takes approximately ninety days. Furthermore, it is the only crop that needs to be transplanted and needs abundant water during the growing phase. 

Applying novel technologies is vital for increasing productivity and efficiency in monitoring paddy crops. Currently, remote sensing information, both retrieved from satellite or unmanned aerial vehicles (UAV), is applied in a wide variety of situations, such as paddy mapping [14,22,23,24,25,26,27,28], yield estimation [29,30,31], crop simulation [32,33,34], damage assessment after flooding [7,10,11,12,13,17,18,19,35,36,37,38], and drought periods [4,5,6,39]. This has been incremented with the ever-increasing stream of available remote sensing data, leading to a continuously increasing need for performance tools to handle all of this information. In this context, machine learning (ML)-driven solutions are shown to be highly beneficial in retrieving data from satellite imagery [40,41].

The presence of ML is significantly increasing in mapping paddy crops [42,43,44,45] or classifying different types of land cover [28,46] and is expected to keep gaining popularity over traditional methods such as thresholding [13,23,47,48,49].

Paddy crop mapping approaches are diverse and depend on the methodology used. These approaches include traditional remote sensing methods such as vegetation indices or a time-series analysis; machine learning techniques, including supervised and non-supervised algorithms; and modelling paddy crop production using satellite imagery. Data sources include optical and radar-based satellite imagery and UAV-carried sensors.

To create a benchmark to inspire new research on this topic, this study reviews different approaches and techniques for mapping paddy crops and distinguishing each stage of crop maturity that have been used since 2010. The content includes a detailed approach to the methods used to map paddy crops and a detailed description of the incidence of ML in the last years, its impact, and the different techniques used to identify the different crop maturity stages and other general statistics indicators.

This paper aims to get an accurate overview of the research background by reviewing the state-of-the-art in identifying paddy crops and their stages by exploring existing remote sensing datasets, methodological approaches, ML techniques, and parameters applied. The systematic literature review was executed using PRISMA guidelines to accomplish this goal. 

## 2. Materials and Methods

Systematically reviewed publications indexed in Thomson Reuters’ Web of Science (https://www.webofscience.com) (accessed on 22 October 2022) and Elsevier’s Scopus (https://www.scopus.com/) (accessed on 22 October 2022) to identify scientific work relevant to cropland monitoring using remote sensing with or without ML have been considered. As noticed in previous explorations, other databases were not considered since they could significantly increase the number of duplicate articles. Preferred Reporting Items for Systematic Reviews and Meta-analyses (PRISMA) have been used as a guideline to perform this review [50]. PRISMA’s methodology has been approved, applied, or widely used in research articles of different areas of expertise [51,52,53,54]. 

One query was generated with combinations of different thematic areas and keywords. Those relations were (a) paddy or rice, (b) identification or mapping, (c) assessment, and (d) remote sensing or satellite imagery or ML or deep learning. Those keywords were added to ensure all papers were within those large thematic areas and the ones talking about those common issues. After thematic areas were identified, a query was developed using the following combination: (“paddy” OR “rice”) AND (“identification” OR “mapping”) AND (“assessment”) AND (“remote sensing” OR “satellite imagery” OR “machine learning” OR “deep learning”). Keywords were set to appear either in the title, abstract, or in the keywords defined in each paper.

Only papers published from 2010 up to 20 October 2022 were considered. In addition, papers included in peer-reviewed journals and conference articles were also considered for screening. 

**Figure 2:**
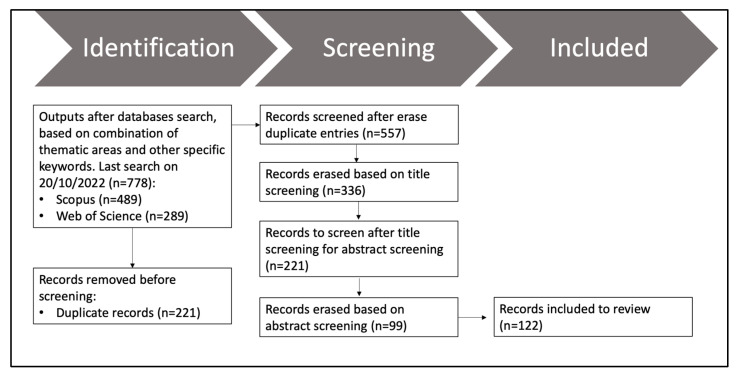
Workflow followed by authors under PRISMA guidelines with all filters per step to identify suitable papers for review.

As PRISMA guidelines suggest, the approach was broken down into three phases: identification, screening, and inclusion (Figure 2). 

In the identification phase, 779 articles were selected to initiate the process. The removal of duplicate articles is considered in this phase. After removing the duplicated articles, a total of 557 articles were eligible for the screening phase. 

During the screening phase, there are two sub-steps for article filtration: title and abstract. Based on the title screening, 336 articles were excluded bringing the number down to 221 eligible for abstract screening. From 221 articles, 98 titles were erased after the abstract screening. The criteria followed in both title and abstract screening to exclude articles: articles that did not include any remote sensing as a source (e.g., digital camera pictures taken on the ground), articles in which rice is not a primary research goal, and articles using sensors other than multispectral and radar (e.g., hyperspectral).

In the inclusion phase, 122 articles were eligible to retrieve and analyse relevant information. Table 1 shows information obtained for each of the articles. In the first instance, general information was collected to monitor the year and journal (peer-reviewed and conference proceedings) along with other standard statistics such as title, author, digital object identifier (DOI), and keywords. In a second category, geographic information was collected to determine the papers’ distribution. In a third category, information was gathered about the tools used in addition to the accuracy assessment and if the paper uses machine learning. The last group is about the approach used for each of the papers based on four options: remote sensing-based approach, machine learning, modelling, and others. In addition, methodologies on how the authors extracted information about crop maturity were gathered in four stages: nursery, transplanting, reproductive, and harvesting phases. Finally, the main constraints identified in the articles in their methodologies were collected.

## 3. Results

### 3.1. Year of Publication and Presence of Machine Learning

A hundred twenty-two articles published between January 2010 and 20 October 2022 were analysed. Overall, there is an increasing pattern in the number of articles published related to this topic. Except for a significant hike in 2014, a smooth increase in publications until 2017 is observed. From 2017 there is a spike in the number of published articles, with a drop in 2021. The years 2020 [5,8,9,26,43,47,55,56,57,58,59,60,61,62,63,64,65,66,67,68] and 2022 (until 20 October) [2,6,18,28,39,42,44,49,69,70,71,72,73,74,75,76,77,78,79,80] showed a higher number of articles published, 20 per year, which represents 16% of the total, 32% combined. 

**Figure 3:**
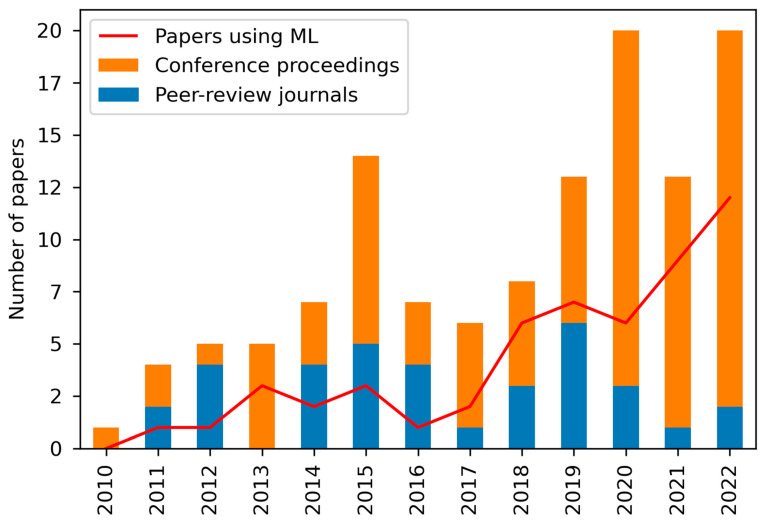
Bar plot showing the distribution of conference proceedings papers and peer-review journal papers across the years. Line plot showing the number of papers that were using ML per year.

Out of all papers, 43% of the papers have used machine learning techniques as part of the workflow proposed to map or classify paddy crops. As shown in Figure 3, ML has increased its presence across the years, being the methodology chosen for 60% of the papers in 2022.

### 3.2. Journals and Conference Proceedings

Articles from peer-reviewed journals and conference proceedings have been considered, representing 72% of the total for peer-reviewed journals and 28% of conference proceedings. 

**Figure 4:**
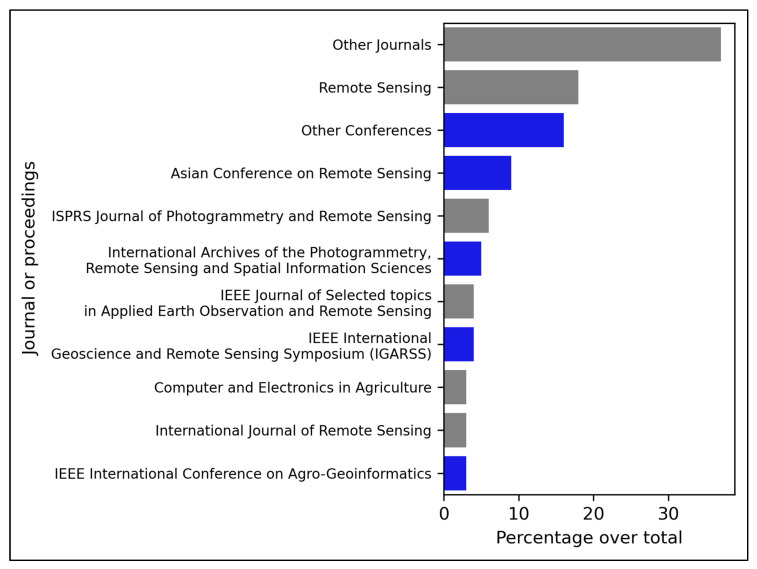
Contribution of journals in percentage to the total number of articles. In grey are peer-reviewed journals, and in blue are conference proceedings.

Amongst peer-review journals, Remote Sensing gathers most publications (Figure 4). Twenty-two articles have been published in this journal, representing 18% of the total publications [2,5,8,12,13,14,18,26,28,29,46,47,61,65,67,69,74,81,82,83,84,85].

The International Society for Photogrammetry and Remote Sensing (ISPRS) Journal of Photogrammetry and Remote Sensing published six papers [9,15,24,86,87,88], representing 6% of the total publications. Institute of Electrical and Electronics Engineers (IEEE) Journal of Selected Topics in Applied earth observation and Remote Sensing (J-STARS) published a total of five papers representing 4% of the total publications [17,56,70,89,90]. Computer and Electronics in Agriculture [58,79,91,92] and International Journal of Remote Sensing [6,43,93,94] have published four papers each, representing 3% of the total publications. All the journals representing less than 3% of the whole were considered as “Other journals”, which are 37% of the total publications.

Regarding conference proceedings papers, the Asian Conference on Remote Sensing had a higher contribution, with eleven articles [62,95,96,97,98,99,100,101,102,103,104] representing 9% of the total publications. The main reasons for those results could be as it was a remote sensing-focused conference and the high interest and relevance of paddy production in Asia, as is represented in Section 3.3 of this article. 

IEEE International Geoscience and Remote Sensing Symposium (IGARSS) published five articles representing 4% of the total publications [16,37,105,106,107], and the IEEE International Conference on Agro-Geoinformatics published four papers, representing 3% of the total publications [19,108,109]. Following the same pattern as with the peer-reviewed journals, all conference proceedings representing less than 3% were gathered in one category called “Other conferences”, representing 16% of the total publications.

### 3.3. Location

**Figure 5:**
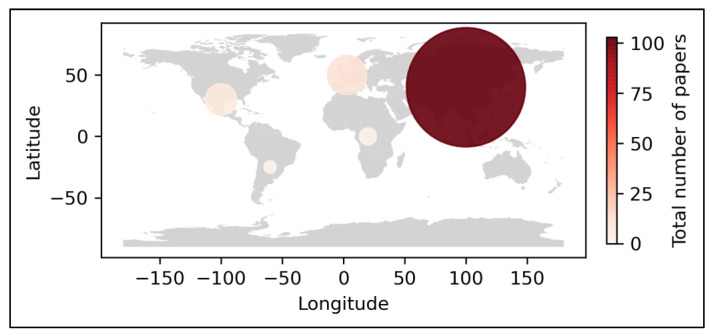
Distribution of the papers geographically.

The hundred twenty-two articles were distributed over five continents following an unequal distribution. A significant number of studies were conducted on the Asian continent, with a total of 101 (Table 2), representing 83% of the total publications, followed by Europe with twelve (9%), North America with six (5%), Africa with two (2%), and finally, both South America and other studies [93] carried out in more than one region with one (1%) each (Figure 5).

The countries represented are twenty-nine in total. China (38) is the most represented, followed by India (14), Thailand (9), and the Philippines and Indonesia (6). 

### 3.4. Data Source and Sensors

#### 3.4.1. Data Source

**Figure 6:**
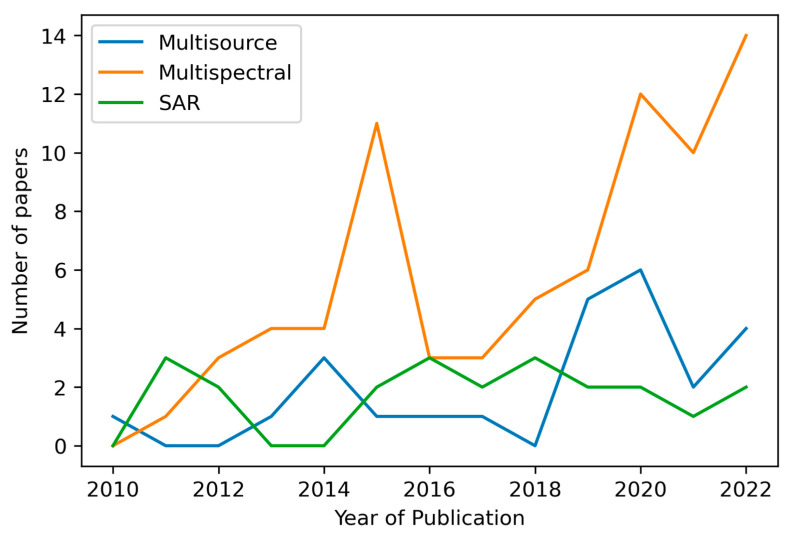
Sensor type of publications.

Multiple sensors have been used across all the articles in the review. Thus, this article has categorised the type of sensor rather than the number of sensors for each paper. Three distinct categories have been identified (Figure 6): (a) multisource, which includes two or more different images coming from different sensors and different categories (optical and radar); (b) multispectral, which includes one or more different images coming from different sensors but same category, in this case, optical, and (c) radar, which includes one or more different images coming from different sensors but same category, in this case, synthetic aperture radar (SAR).

A strong dominance of multispectral-based solutions is the solution adopted by seventy-five articles, representing 62% of the total. The multisource-based solution was adopted by twenty-five articles, representing 20% of the total publications. Finally, twenty-two radar-based solution articles represented 18% of the total publications (Figure 6) [138,139].

#### 3.4.2. Sensors

The literature review also reveals a wide variety of sensors used. MOderate Resolution Image Spectroradiometer (MODIS) was used in forty-seven cases representing 28% of the total publications (Table 3). MODIS sensor operates both the Earth Observation System’s Terra and Aqua satellites launched by the National Aeronautics Space Administration (NASA) in 1999 and 2002 [138].

**Figure 7:**
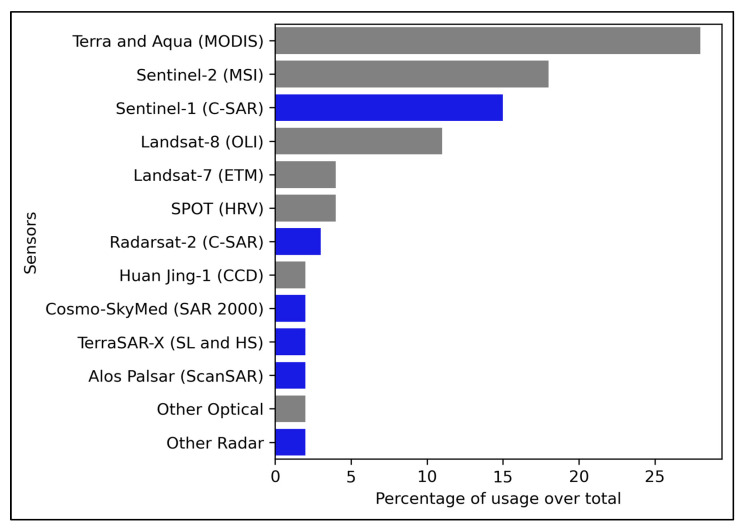
List of different satellites, with the sensors in parenthesis, and their presence in the re–viewed articles (optical sensors are shown in grey and radar sensors in blue).

Multi-Spectral Instrument (MSI) involved in European Space Agency’s (ESA) Sentinel-2 is the second most used, with twenty-nine articles, representing 18% of the total publications. C-SAR active sensor carried in the ESA’s Sentinel-1 is the third with more appearances, with twenty-eight, representing 17% of the total publications. Active (or radar) sensors produce their wavelength and then record the reflection of this wavelength after interacting with the Earth. Sentinel-1 is also ranked as the main preference for satellites carrying radar sensors. The Operational Land Imager (OLI) sensor carried in USGS’s Landsat-8 with twenty appearances, represents 12% of the total publications. Other varieties of sensors were identified but with less than ten appearances were grouped in “Other optical” and “Other radar” categories (Figure 7).

### 3.5. Approach

The 122 articles identified approaches depending on how remote-sensed imagery was used to get solutions: (a) remote sensing-based approach, (b) machine learning, (c) model, and (d) others. The remote sensing-based approach includes all articles that used different band operations (e.g., vegetation indices), time-series analysis, and algorithms where it is not included in ML. The ML approach includes all the articles that used machine-learned techniques (including deep learning) to get their solutions. Those articles that used remote-sensed imagery to feed an own-developed model or existing modelling software fall into a model category. Finally, within the “other” category, all the papers with a combination of approaches were included.

The remote sensing-based approach was a preferred technique to solve problems in sixty-six of the cases, 54% of the total solutions. ML approach, with forty-three appearances, represented 35% of the total solutions. The model approach was used in ten articles, representing 8% of the total solutions. The last category, categorised as “other”, with three papers identified, representing 2% of the total solutions [24,83,131]. The “other” category combined the remote sensing-based and machine-learning approaches.

Within the remote sensing-based approach, considering vegetation indices, the Normalized Difference Vegetation Index (NDVI) [139] is the vegetation index used more frequently, with a total of thirty appearances (Table 4), representing 30% of the total solutions. The contrast between the red channel absorption and the near-infrared reflection of the electromagnetic spectrum gives relevant information for crop monitoring applications [140]. With twenty-five appearances, the Enhanced Vegetation Index (EVI) [140] is the second most used vegetation index representing 25% of the total solutions. EVI added reflectance in the blue channel, helping to minimise soil background noise and atmospheric influences [140]. Land Surface Water Index (LSWI) [141] is the third most used, with fourteen appearances, representing 14% of the total. 

**Figure 8:**
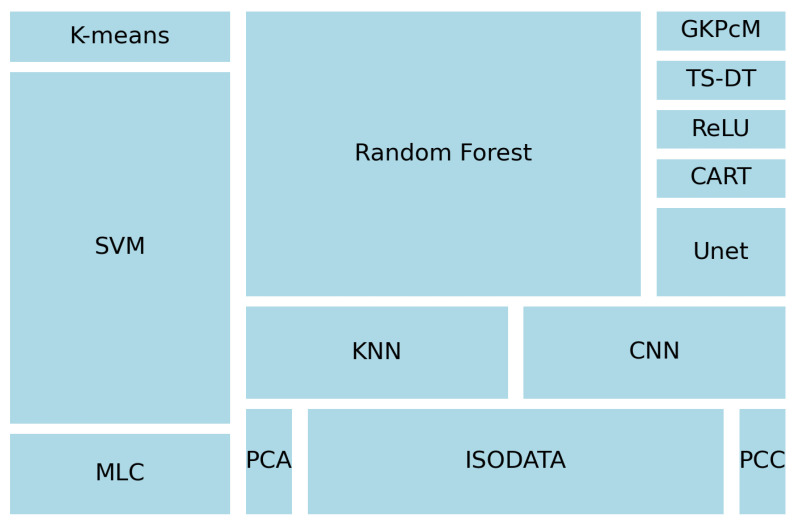
Treemap showing ML algorithms identified in the review and the frequency of appear–ance.

Looking into the ML algorithms, random forest, with seventeen appearances, is the most used algorithm, followed by support vector machine (SVM) with twelve appearances and isodata with seven (Figure 8). ML algorithms could be classified into two larger groups: supervised and unsupervised. Supervised ML is characterised by using labelled datasets to train algorithms to classify images. On the other hand, unsupervised ML attempts to identify hidden patterns to label a dataset. If ML algorithms are gathered for consideration in this classification, supervised ML represents 80% (47) of the solutions and is preferred against unsupervised ML 20% (12).

Focusing on the modelling approach, researchers used satellite imagery to feed yield crop software models such as ORYZA [30,31,47,62,104,111,131] or MAPscape-RICE [11,30,31,126]. ORYZA model (https://www.irri.org/oryza, accessed on 23 March 2023) is a software belonging to the International Rice Research Institute (IRRI) aiming to model and compare the performance of simulated numbers with in situ observations. MAPscape-RICE is software that detects rice areas, season start, and planting dates by ingesting post-processed remotely sensed imagery [24,26,67,83,116,131].

Falling into the “other” category, often the approach followed used a remote sensing-based method with the application of vegetation indices to get phenology data for detecting paddy rice and using this information to feed a machine learning-based algorithm to map paddy fields or compare accuracies [24,83].

### 3.6. Study of Paddy Stages

Thirty-three articles have studied how to identify each paddy crop stage, depending on its maturity. Four different maturity stages were considered [21]. The timeline of each of the stages could be different depending on other factors such as the type of rice grain, rice crop dynamics, or the area in which it has been seeded or the type of soil underneath [8,23,29,90,110,115,118].

#### 3.6.1. Multispectral Approach

A total of twenty-two articles used vegetation indices to extract this information. Retrieved from multispectral imagery, Normalized Difference Vegetation Index (NDVI), the Enhanced Vegetation Index (EVI), and Land Surface Water Index (LSWI) are the most used vegetation indices to determine those paddy stages being used in eleven [16,43,49,88,91,108,115,116,117,127,135], eight [16,25,81,83,86,114,117,135], and seven publications [16,24,25,86,114,124,135], respectively. They combine different ways to extract this information. Some authors determine the end of the nursery stage and the start of transplanting by the combination of the descending trend of chlorophyll-based indexes (NDVI, EVI) and the rapid increase in LSWI [16,25,86,114,135]. Other authors have used the same approach but combined vegetation indices like Bare Soil Index (BSI) with LSWI [24]. Others, after the use of the coefficient of variation of LSWI, determined planting stages [124]. Others combined several vegetation indices by looking for correlations and peak or bottom measures of each to define different steps. To determine the nursery with high BSI values following the vegetative phase, a combination of a fast drop in BSI and a rapid increase in LSWI [24] at the initial days of the stage, followed by a rapid rise in NDVI [88,91,115,116,127], EVI [16,25,81,83,86,117,135] and Leaf Area Index (LAI) [136] are assessed. To identify the reproductive phase, NDVI reaches its peak [24,49,88,91,108,115,116,127] along with EVI [24,49,81,83,88,91,108,115,116,117,127] to identify the harvest phase, and there is a marked rise in Plant Senescence Reflectance Index (PRSI) index combined with a severe decrease in NDVI and EVI due to loose of chlorophyll content in paddy [24] and with LSWI at its lowest value [25,124].

All these articles presented good accuracy results, obtaining an interval of 70–99% overall accuracy [24,25,43,49,83,86,90,108,117,124,135] for those validated against national land cover maps or a correlation above 80% to those validated against national official statistics bureau [81,86,91]. However, some constraints were reported. All have in common that the effects of cloud cover contamination in the pixels could impact the accuracy. In addition to this general constraint, some specific limitations were pointed out: (a) the misclassification with wetlands in the transition from nursery to vegetative phase [24,124] due to the high presence of water in paddy fields; (b) the misclassification or salt-and-pepper misclassification due to the use of coarse resolution sensors, especially in the case of MODIS [81,115]; and (c) the potential problems to distinguish the different paddy stages in other conditions such as rainfed paddy rice regions [124].

#### 3.6.2. Radar Approach

A total of twelve articles used radar sensors to identify paddy stages’ information. Most of them analysed the time series of SAR imagery in their study area. The pattern followed by SAR imagery is normally marking medium level backscatter value in the nursery stage due to the rough surface [123] and marks its lower backscatter signal value during the transition between the nursery and transplanting phase due to the high volume of water present and the low quantity of vegetation, reaching its higher backscatter value within the reproductive phase, due to the high amount of paddy cover and its texture. Finally, at the harvesting stage, the backscatter value slightly decreases from its peak [14,23,26,44,48,55,79,96,104,106,113,123].

Those articles presented 77 to 99% overall accuracy when confronted against national land cover maps [14,26,44,79,104,113,123] or good correlation values when validated against official governmental statistics between 0.87–0.96% [79,113]. However, as in the multispectral approach, some constraints could arise and could affect the result: (a) misclassification with other land covers like shrubs and herbs in presence of heavy rain [79] or sandbars of rivers, highly recommending the use of water mask [48]; (b) gaps on Sentinel-1 acquisition [23]; and (c) limitations on timing ground truth data acquisition with SAR coverage [55].

### 3.7. Accuracy Assessment Techniques

A total of sixty-seven articles used the overall accuracy (OA) test to evaluate the validity of their outputs (Table 5). The OA tells what proportion of the output was mapped correctly. In most cases, authors also calculated the user’s accuracy (UA), which is the accuracy from the point of view of the map user, and the producer’s accuracy (PA), which is the map accuracy from the map maker’s point of view. This technique was chosen by the authors when they had digital data as reference and to validate with an existing official land cover map [2,4,10,11,15,22,23,30,56,65,85,86,98,99,103,119,135] and ground-truth data acquired [2,14,22,25,73,77,81,83,84,87,95,101,105,112,113,117] or to sample using photointerpretation of a very high-resolution image (e.g., Google Earth imagery) [79].

A total of twenty-nine articles used the correlation coefficient to validate their results. It measures the strength and direction of the relationship between two variables. All authors have used it when there are official statistics either listing the total amount of hectares of paddies or the production.

Other validated accuracy techniques also used by the authors are root mean square error (RMSE), median absolute error (MAE), and omission error (OE).

## 4. Discussion

Most of the work performed is concentrated in Asia. Some regions are very few or not present in this scientific work. Even though Asia accounts for most of the rice consumption per capita in the world at above 75%, other regions such as Africa, Europe, Oceania, and North America are expected to increase their consumption [1]. Africa is expected to contribute more to rice production than other cereals due to major consumption and the expected decrease in harvested areas in China [1]. It is essential to get suitable solutions to monitor rice crops in other regions, especially in countries where there can be an expected growth in population, production, and consumption. Those solutions should go towards monitoring rice production and assessing the impact of how different threats like climate change, food security, spatial planning, or land management affects it. 

The diversity of data sources reveals the wide variety of instruments and missions available. The dominance of the multispectral-based approach could be due to (a) more extensive data available coming from different satellite missions since 1970′s first Landsat mission [142], (b) a large number of free-of-cost optical sensors with an improvement in their resolution, especially in recent years available, and (c) the easy and straight-forward interpretation of data [143].

Optical sensors are the most used by the authors because of their more straightforward processing and the ease of interpretation of their images, which are much closer to the perception detected by the human eye. The results show that among them, MODIS has been the most used due to the broader coverage per scene, the availability of satellite images for the whole study period considered, and mainly because of its temporal resolution, and the Terra- and Aqua-MODIS instruments observe the entire surface of the Earth every one or two days. However, other sensors, such as Sentinel-2 MSI and Landsat-8 OLI, can barely reach temporal resolutions of five days in the case of the combined use of the S2 constellation or sixteen days in the case of Landsat 8. However, with the launch of Landsat 9 on 27 September 2021, the revisit period is reduced by half, so an increase in the use of both systems is expected in the coming years. Although the latter sensors, due to their better spatial resolution, 10 m for Sentinel-2 MSI and 30 m for Landsat-8 OLI, as opposed to the coarser resolution of MODIS, between 250 and 1000 m, depending on the spectral bands considered, have traditionally been used to study rice fields occupying small areas, and their images, despite being freely available, relatively more difficult to download and manipulate due to their weight, from servers such as Earth Explorer of the USGS (https://earthexplorer.usgs.gov/, accessed on 31 March 2023) or Sentinel—Copernicus Open Access Hub. Nowadays, with the creation of cloud platforms such as Google Earth Engine [144], the whole dataset can be easily managed and efficiently post-processed, due to higher resolution images are already ready to be used to cover large areas around the planet. 

Therefore, their use will increase in the coming years. However, some constraints, such as cloud contamination [5,8,43,57,59,60,65,81,85,89,108,130,136], are all common. Cloud contamination could be easily avoided using radar data. Radar presents more complexity to process. However, in platforms like GEE, Sentinel-1 C-SAR data can be used in the analysis-ready format. Sentinel-1 C-SAR sensor images are already the third with a higher presence in this study. It provides around 10 m spatial resolution and six days temporal resolution. Thus, increasing use of radar data can be foreseen shortly, even though it will not replace optical sensors.

The remote sensing-based approach is the most commonly used by the authors. Optical sensors traditionally use those techniques. It has been used for a long time and involves different procedures. Furthermore, with new satellite data produced by modern high-resolution sensors, techniques like time-series analysis and especially ML have more data to gather, keeping higher accuracies.

Although the second most used, ML has expanded its emergence in this topic since 2016. Figure 1 shows a spike in the use of ML from 2016 to nowadays, being the preferred approach by 60% over 2022 (until 20 October). In addition, in 80% of the cases, supervised ML has been used as the preferred option. This is due to authors either using pre-existing data to train their classifier or having picked ground-truth data. ML is the preferred approach in the upcoming years. However, when new research focuses on remote areas (e.g., some areas of Africa), where there is a need for more data, and it is difficult to collect ground-truth data, unsupervised classifiers could gain presence. The first option for authors was to use the random forest algorithm. The ability to handle large datasets along with the ability to handle noisy or irrelevant features, the possibility to allow numerical and categorical inputs, and the fact that it does not require feature normalization could explain why it is the favourite for the authors. SVM also shows a high use by the authors. The good behaviour, especially when datasets are not large, makes it a good ML algorithm to consider when training and test datasets are not large.

Researchers used a multispectral or radar approach for other paddy stage identification. Using the multispectral approach, it is possible to retrieve phenological information by performing band mathematics (e.g., vegetation indices) and classification by considering the spectral signature. On the other hand, information regarding the texture and altitude of the crop could be retrieved using a radar approach. Both methods present similar accuracies but various constraints. The multispectral approach presents cloud contamination [43,81,108,115], mixed pixel issues [81,83], and misclassification with wetlands [24,124] as the most common constraints. The radar approach presents constraints of misclassification with river sandbars [124], herbs, and shrubs [79]. Therefore, there is a space for further research to adopt multisource solutions to benefit from multispectral and radar approaches’ advantages and avoid some of the limitations identified.

The accuracy assessment methodology is essential to validate the work performed correctly. However, the chosen technique is diverse depending on the data available for validation. When there is either ground-truth data or image support, created through photo-interpretation or existing in a land cover map, authors used OA, PA, and UA methodologies to validate their results. On the other hand, when national statistics about paddy acreage or production were available, the authors in its majority used the correlation coefficient approach to compare its results against statistics.

## 5. Conclusions

This review covered remote sensing papers published between 2010 and 20 October 2022, identifying paddy rice crops and each stage to retrieve information about crop maturity.

The continuous growth of the planet’s population, linked with other threads (e.g., the ongoing climate change consequences), puts pressure on the paddy cropping systems. Thus, better dataset monitoring systems and models are required to comprehend better how countries are settled in spatial planning, land management and food security. Therefore, this paper assessed the current state-of-the-art remote sensing solutions in this topic using the PRISMA statement guidelines. This methodology was suitable for quantifying obtained information about several parameters of interest in the subject that will be of high value towards new studies to come.

In a rapidly changing world, it is expected that variables such as climate change, season impact, water limitation, and disease presence, among others, will gain presence to develop more accurate monitoring tools.

It is expected that machine learning usage will gain more terrain against other remote sensing-based approaches due to: (a) ever-streaming opensource data availability and (b) continuous innovation on the domain expertise (e.g., Meta AI segment anything algorithm [145]).

This paper gathered relevant information on identifying each paddy crop stage by presenting multispectral and radar-based solutions. However, by collecting the advantages and disadvantages of multispectral and radar-based solutions, future research could combine them by fusing or using both solutions in a decision-tree schema to maximise the benefits of each method.

Even if valuable and relevant, some proposed future directions could go to differentiate methods for the type of cropping system (rainfed or irrigated) or to quantify the assessment of crop diseases.

## Figures and Tables

**Table 1:** Breakdown of all information collected per each article.

Feature	Data Type	Description
Title	Text	Article title
Author	Text	Authors’ names
Year	Categorical	Year of publication
Journal	Text	Journal of publication
DOI	URL	Article’s DOI link
Keywords	Text	Article’s keywords
Region	Categorical	Continent in which the study was carried out
Country	Text	Country in which the study was carried out
Data source	Text	General type of data source used (e.g., multispectral, multisource and radar)
Sensor	Text	List of sensors used (e.g., Sentinel-2, Sentinel-1, Landsat-8, etc.)
Accuracy assessment method	Text	Accuracy assessment method used (overall accuracy, correlation ($R^{2}$), user’s accuracy, etc.)
Machine learning	Categorical	Shows if machine learning was used in the article, possible answers (Yes/No)
Approach	Text	Approach on how remote sensing data was used (e.g., remote sensing-based approach, machine learning, model input, etc.)
Stage of maturity	Text	It classifies the method used to classify/extract/map/identify paddy in various stages of maturity (nursery, vegetative phase, reproductive phase, and harvesting phase)
Main constraints	Text	Main constraints identified by the article regarding the methodology or sensor used

**Table 2:** Table showing the location of the studies by continent.

Location	Literature Number	Reference
Asia	101	[2,4,5,6,7,8,9,10,12,13,14,15,16,17,18,19,22,25,26,28,30,31,36,37,38,39,42,43,44,45,46,48,49,55,56,57,58,59,61,62,65,66,69,71,72,74,75,77,78,79,80,81,82,83,84,85,86,87,90,91,92,94,95,96,97,98,99,100,101,102,103,104,105,108,110,111,112,113,114,115,116,117,118,119,120,121,122,123,124,125,126,127,128,129,130,131,132,133,134]
Europe	12	[11,23,29,35,63,70,76,88,106,107,135,136]
North America	6	[47,73,89,109,137]
Africa	2	[60,68]
South America	1	[64]

**Table 3:** Table showing the most represented satellites and sensors in the review.

Satellite (Sensor)	Literature Number	Reference
Terra and Aqua (MODIS)	47	[2,4,5,6,12,13,14,15,17,25,28,36,39,56,59,64,65,67,74,75,77,80,81,82,85,86,87,88,89,98,108,110,112,114,116,117,118,129,132]
Sentinel-2 (MSI)	29	[5,8,9,10,18,24,26,29,42,45,46,60,63,66,67,68,69,71,72,76,80,92,104,107,108,130,133,134,136]
Sentinel-1 (C-SAR)	28	[9,10,11,18,22,23,26,31,44,45,46,47,55,60,62,67,69,71,79,80,90,101,102,103,104,119,126,130]
Landsat-8 (OLI)	20	[2,6,7,11,13,19,39,43,47,57,65,72,73,77,80,85,104,108,109,122]

**Table 4:** Table showing the most used vegetation indices represented in the review.

Vegetation Index	Formula	Literature Number	References
Normalized Difference Vegetation index (NDVI)	$\frac{\left(NIR-RED\right)}{\left(NIR+RED\right)}$	30	[2,4,5,7,13,16,17,24,49,61,63,64,65,76,84,86,88,91,99,100,108,112,117,125,127,129,134,135]
Enhanced Vegetation Index (EVI)	$2.5\;\frac{\left(NIR-RED\right)}{\left(\left(NIR+6RED-7BLUE\right)+1\right)}$	25	[4,8,11,12,13,16,17,25,36,37,59,65,66,81,82,83,86,89,98,100,108,114,117,134,135]
Land Surface Water Index (LSWI)	$\frac{\left(NIR-SWIR\right)}{\left(NIR+SWIR\right)}$	14	[8,16,25,36,65,66,86,89,114,124,135]

**Table 5:** Table showing the most represented accuracy assessment technique and the number of times used in the review.

Accuracy Assessment Technique	Literature Number	Reference
Overall Accuracy (OA)	67	[2,7,8,10,11,12,14,15,18,19,22,23,24,25,26,30,31,43,44,49,56,57,58,62,65,67,69,70,72,73,74,75,77,79,80,81,83,84,85,86,87,89,90,93,95,98,99,100,101,103,104,105,108,109,112,113,117,118,119,122,123,124,125,128,135,137]
Correlation Coefficient	29	[4,6,13,14,18,25,28,29,39,68,73,75,77,79,81,82,84,85,86,87,91,94,98,100,110,112,119,126,129,132]
Root Mean Square Error (RMSE)	5	[59,75,120,132,136]
Omission Error (OE)	4	[11,45,110,135]
Median Absolute Error (MAE)	3	[59,120,132]

## Data Availability

The data presented in this study can be made available upon request from the authors.
